# Phenotypic Characterization of Human Monocytes following Macronutrient Intake in Healthy Humans

**DOI:** 10.3389/fimmu.2017.01293

**Published:** 2017-10-23

**Authors:** Awad Alshahrani, Abdalmalik Bin Khunayfir, Mohammed Al Rayih, Hasan Al Sayed, Abdullah Alsadoon, Mohammed Al Dubayee, Mahmoud Zahra, Yousof Alrumayyan, Maha Al Zayer, Amre Nasr, Ahmad Aljada

**Affiliations:** ^1^Department of Basic Medical Sciences, King Saud bin Abdulaziz University for Health Sciences, Riyadh, Saudi Arabia; ^2^King Abdullah International Medical Research Center (KAIMRC), Ministry of National Guard Health Affairs (MNGHA), Riyadh, Saudi Arabia

**Keywords:** mononuclear cells, monocyte polarization, monocytes subsets, macronutrient intake, whey proteins

## Abstract

**Background:**

Three subsets of human monocytes in circulation have been identified and their characterization is still ill-defined. Although glucose and lipid intakes have been demonstrated to exert pro-inflammatory effects on mononuclear cells (MNCs) of healthy subjects, characterization of monocytes phenotypes following macronutrient (glucose, protein, and lipid) intake in humans remains to be determined.

**Methods:**

Thirty-six healthy, normal weight volunteers were recruited in the study. Subjects were randomly assigned into three groups, each group consisting of 12 participants. Each group drank equal calories (300 kcal) of either glucose or lipids or whey proteins. Each subject served as his own control by drinking 300 mL of water 1 week before or after the caloric intake. Baseline blood samples were drawn at 0, 1, 2, and 3-h intervals post caloric or water intakes. MNCs were isolated, and the expression levels of different cluster of differentiation (CD) markers (CD86, CD11c, CD169, CD206, CD163, CD36, CD68, CD11b, CD16, and CD14) and IL-6 were measured by RT-qPCR.

**Results:**

Equicaloric intake of either glucose or lipids or whey proteins resulted in different monocyte phenotypes as demonstrated by changes in the expression levels of CD and polarization markers. Whey proteins intake resulted in significant mRNA upregulation in MNCs of CD68 and CD11b at 1, 2, and 3 h post intake while mRNA of IL-6 was significantly inhibited at 1 h. Lipids intake, on the other hand, resulted in mRNA upregulation of CD11b at 2 and 3 h and CD206 at 1, 2, and 3 h. There were no significant changes in the other CD markers measured (CD86, CD163, CD169, CD36, CD16, and CD14) following either whey proteins or lipids intakes. Glucose intake did not alter mRNA expression of any marker tested except CD206 at 3 h.

**Conclusion:**

Macronutrient intake alters the expression levels of polarization markers in MNCs of human subjects. A distinct population of different monocytes phenotypes may result in human circulation following the intake of different macronutrients. Further studies are required to characterize the immunomodulatory effects of macronutrients intake on monocytes phenotypes and their characteristics in humans.

## Introduction

In recent years, three subsets of human monocytes in circulation have been identified based on the expression of the surface markers CD14 and CD16 (1). The specific roles for these subsets in homeostasis and inflammation are still unclear. Additionally, the characterization of these subsets of human monocytes is still ill-defined and in its infancy. The classical monocytes represent the major population of human monocytes (90%) and express high levels of CD14 and lack CD16 expression (high CD14 and no CD16). The other remaining 10% of human monocytes population is subdivided into the intermediate subset (high CD14 and low CD16) and the non-classical subset (relatively lower CD14 expression and high CD16) (1).

Macrophages differentiate from circulating monocytes. The dynamic recruitment of circulating monocyte into peripheral tissue occurs in response to chemical signals linked to infection, necrosis, or trauma, effectively mobilizing immature monocytes from the circulation to tissue and promoting their differentiation into mature tissue-specific macrophages (2). Mature tissue-specific macrophages express receptors that interact with their local chemical environment. This includes fate-determining growth factors, pro-inflammatory cytokines, and microbial components. Cytokine–receptor interactions dramatically alter macrophage physiology, signaling the expression of different surface receptors, and the secretion of pro-inflammatory mediators and cytokines. During inflammation, specific reciprocal interactions between macrophages and T and B cells (*via* cytokines–receptor interaction) promote further activation, enhanced regulation, and the acquisition of specific functional phenotypes (3). There are three main macrophage phenotypes: a pro-inflammatory (M1), an anti-inflammatory pro-tissue (M2), and metabolically activated (MMe) macrophage phenotypes (3, 4). The classically activated macrophages are triggered by Th1-derived interferon-gamma (IFNγ) and lipopolysaccharides (LPS) (5, 6). LPS and IFNγ polarize resident macrophages (M0) toward the M1 subtype, which secretes pro-inflammatory cytokines such as tumor necrosis factor (TNFα), IL-1, and IL-6. These cytokines are implicated in initiating and sustaining inflammatory function (7). M1 macrophages, therefore, mediate intracellular killing of pathogens and produce microbicidal reactive nitrogen and oxygen intermediates that promote inflammation. Furthermore, M1 macrophages express unique surface markers such as CD80 and CD86. On the other hand, interleukin-4 (IL-4) and IL-13 induced different or “alternative” activation for macrophages, largely by inhibiting the expression of the major surface markers usually found in classically activated macrophages (8). IL-4 and IL-13 induce macrophage polarization into the M2 subtype, which produces anti-inflammatory cytokines such as IL-10. M2 macrophages are characterized by strong IgE response and are involved in fungal and parasitic infections and tissue remodeling (3). These two phenotypes metabolize arginine differently. M1 macrophages convert arginine to nitric oxide (NO), whereas M2 macrophages convert arginine to ornithine (9, 10). NO has microbicidal functions and can damage lipids, proteins, and DNA and inhibits cell division, while ornithine stimulates cell division and wound healing (11, 12). This finding led to the consensus of the pro-inflammatory versus anti-inflammatory properties of M1/M2 macrophages.

Macronutrient intake has been shown to induce inflammation (13). Glucose and saturated fat (cream)-induced reactive oxygen species (ROS) generation in mononuclear cells (MNCs), which subsequently could lead to lipid, protein, and DNA damage (14, 15). A mixed meal from a fast food chain also induces oxidative stress and inflammation at the cellular and molecular level (16). These data suggest an M1-like monocytes form following macronutrient intake. Interestingly, Kratz et al. recently identified a distinct population of metabolically activated macrophages (MMe) in adipose tissue, following glucose activation (4). MMe do not express the classic markers of M1 cells, suggesting that macrophages might express different phenotypical markers when metabolically stimulated (by glucose, insulin, or palmitate). In this study, we examined monocyte polarization following equicaloric intake of macronutrients (glucose, whey proteins, and lipids) in healthy lean subjects. We hypothesized that different macronutrients could induce different immunomodulatory effects resulting in distinct metabolically activated monocytic phenotypes similar to MMe.

## Materials and Methods

### Subjects

Thirty-six normal healthy adult volunteers (male: 35, female: 1; age range: 20–25 years; mean: 21.4 ± 0.18 years; mean ± SEM) of normal weight (BMI-glucose group: 21.5 ± 0.59 kg/m^2^; BMI-proteins group: 22.4 ± 0.59 kg/m^2^; BMI-lipids group: 22.0 ± 0.46 kg/m^2^; mean ± SEM) were recruited into the study. All were normotensive, had a normal lipid profile, normal renal, and liver function tests, and were not on any medications. The 36 participants were randomly assigned by the primary investigator (PI) following simple randomization procedure (computerized random numbers) to three different groups; each received one type of macronutrient (glucose, whey proteins, or lipids). Following an overnight fast, a baseline blood sample was taken. Subjects were then given either 300 calories of glucose (NERL Trutol 75) or lipids (90 g whipping cream, 31.5 g fat, 1.7 g protein, and 2.25 g carbohydrate) or protein [isopure unflavored whey proteins isolate (WPI) powder containing 26 g per serving of 100% WPI, stripped of fat, carbs, fillers, sugars, and lactose] solution over 5 min. Cream and protein preparations were diluted with water up to 300 mL solutions. Further blood samples were obtained at 1, 2, and 3 h after the macronutrient intake. Subjects, either 1 week before or after the macronutrient challenge, were given 300 mL of water to drink in the fasting state. Blood samples were obtained before and at 1, 2, and 3 h after water intake as well. Each subject served as his/her own control and was randomly given macronutrient or water intake. All subjects gave their written, informed consent. Institutional Review Board (IRB) of the Ministry of National Guard Health Affairs (MNGHA) approved the study protocol. Recruitment of the subjects was done in February 2016, and the collection of samples was concluded on April 2016. The study was conducted at College of Medicine, King Saud bin Abdulaziz University for Health Sciences, Riyadh, Kingdom of Saudi Arabia.

### Isolation of MNCs

Blood samples were collected in Na-EDTA as an anticoagulant. Fifteen milliliters of the anticoagulated blood sample were diluted with an equal volume of PBS and were carefully layered over 50 mL of Ficoll-Hypaque (50 mL Leucosep Tubes, Greiner Bio-One North America Inc., NC, USA). Samples were centrifuged at 450 ×*g*, in a swing out rotor for 30 min at 22°C. At the end of the centrifugation, MNCs separate out at the top of the RBC pellet. MNC bands were harvested with a pipette, repeatedly washed with PBS. Fifty microliters of Qiagen RNAlater were added to the pellets and samples were then frozen at −80°C.

### Glucose and Insulin Measurements

Whole blood glucose levels were measured using ACCU Check Active Blood Glucose Monitor. Serum insulin concentration was determined using ALPCO insulin ELISA kit (Salem, NH, USA) according to the manufacturer’s protocol. Twenty-five microliters of standards, controls, and samples were added to the microplate wells with the detection antibody. The microplate was then incubated on a microplate shaker at 700–900 rpm followed by washing with Wash Buffer. TMB Substrate was then added, and the microplate was incubated a second time on a microplate shaker followed by stop solution, and the optical density was measured by a spectrophotometer at 450 nm.

### mRNA Quantification by Real-time RT-PCR

Total RNA was isolated using the Ambion Aqueous kit (Ambion). The quality and quantity of the isolated RNA were determined using a The Agilent 2100 Bioanalyzer system. Then, 1 µg of total RNA was reverse-transcribed using first strand cDNA synthesis Kit (Millipore, USA). Real-time RT-PCR was performed with a 7900HT Fast Real-Time PCR System (Applied Biosystems, USA), using 2 µL cDNA, 10 µL 2X Sybergreen Master mix [150 mM Tris, pH 9.2, 40 mM (NH_4_)_2_SO_4_, 5 mM MgCl_2_, 0.02% Tween-20, 0.4 mM dNTPs, 1.25 U Taq Polymerase, 1× Sybergreen] and 0.5 µL of 20 µM gene-specific primers (Table 1). The specificity and size of the PCR products were tested by adding a melt curve at the end of the amplifications, analysis on a 2% agarose gel, and sequencing of the bands. All values were normalized to cyclophylin A and RPL-13. The 2^−ΔΔ^*^CT^* method was used for relative quantification for qRT-PCR experiments (17). Baseline expression levels of markers studied for each subject was normalized to a 0 value. Positive values indicate an upregulation while negative values indicate an inhibition in mRNA expression.

**Table 1:** Primer sequences for all primers used in qRT-PCR.

Primer	Sense (5′→3′)	Anti sense (5′→3′)	Accession number
CD206	TTCGGACACCCATCGGAATTT	CACAAGCGCTGCGTGGAT	NM_002438.3
CD86	CTGCTCATCTATACACGGTTACC	GGAAACGTCGTACAGTTCTGTG	NM_175862.4
CD11b	CAGCCTTTGACCTTATGTCATGG	CCTGTGCTGTAGTCGCACT	NM_001145808.1
CD68	GCTACATGGCGGTGGAGTACAA	ATGATGAGAGGCAGCAAGATGG	NM_001251.2
CD169	CCTCGGGGAGGAACATCCTT	AGGCGTACCCCATCCTTGA	NM_023068.3
IL-6	AATAACCACCCCTGACCCAAC	AATCTGAGGTGCCCATGCTAC	NM_000600.4
CD163	CAGGAAACCAGTCCCAAACA	AGCGACCTCCTCCATTTACC	NM_004244.5
CD36	GCCAAGGAAAATGTAACCCAGG	GCCTCTGTTCCAACTGATAGTGA	NM_001001548.2
CD14	AGCCAAGGCAGTTTGAGTCC	TAAAGGACTGCCAGCCAAGC	NM_000591.3
CD16	ATGTGTCTTCAGAGACTGTGAAC	TTTATGGTCCTTCCAGTCTCTTG	NM_000569.7
RPL13	AACAAGTTGAAGTACCTGGCTTTC	TGGTTTTGTGGGGCAGCATA	NM_000977.3
Cyclophilin A	CCC ACC GTG TTC TTC GAC AT	TTT CTG CTG TCT TTG GGA CCT T	NM_021130.4

### Statistical Analysis

Statistical analysis was carried out using SigmaStat software ver. 3.0 (Jandel Scientific, San Rafael, CA, USA). Fold change in mRNA expression was calculated for qRT-PCR results and analysis was carried out with one-factor ANOVA for the repeated measures using Dunnett’s test for comparisons against the baseline (0 h) for normally distributed data. Dunn’s test was used for the non-parametric data. A *P*-value < 0.05 was used to assess significance for all statistical analyses. Results are presented as mean ± SEM.

## Results

**Figure 1:**
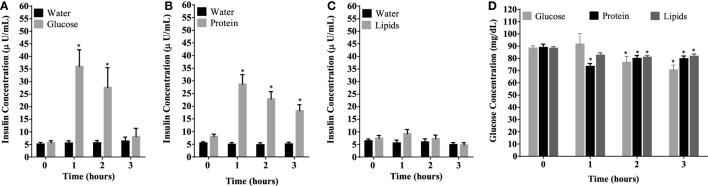
Plasma insulin concentrations (microunits per microliters) **(A–C)** and whole blood glucose concentrations **(D)** at 0, 1, 2, and 3 h post macronutrient intake; *n* = 12 per group; *P < 0.05.

**Figure 2:**
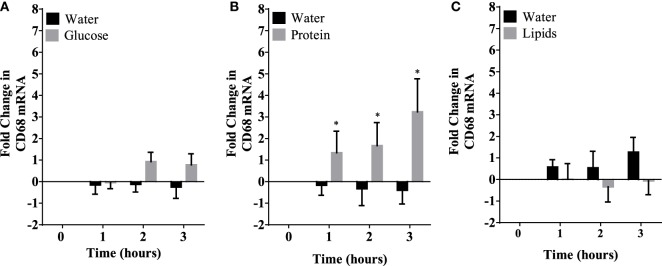
Fold change in mRNA expression of CD68 in mononuclear cells following equicaloric intake of macronutrients. Glucose **(A)** and lipids **(B)** did not change mRNA expression of CD68 significantly while whey proteins intake **(C)** induced mRNA expression of CD68 at 1, 2, and 3 h post macronutrient challenge; *n* = 12 per group; *P < 0.05.

**Figure 3:**
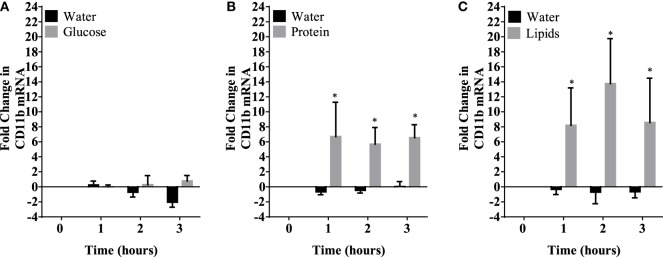
Fold change in mRNA expression of CD11b in mononuclear cells following equicaloric intake of macronutrients. Glucose **(A)** had no effect on CD11b mRNA expression while both whey proteins **(B)** and lipids **(C)** induced mRNA expression of CD11b significantly; *n* = 12 per group; *P < 0.05.

**Figure 4:**
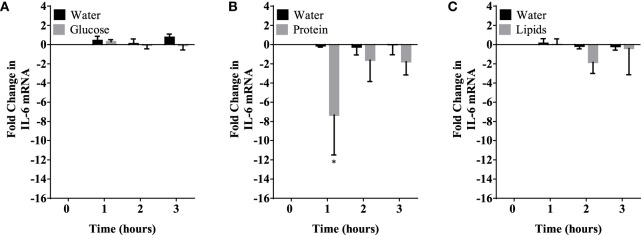
IL-6 mRNA expression levels in mononuclear cells (MNCs) following glucose **(A)**, whey proteins **(B)**, and lipids **(C)**. There was significant inhibition in IL-6 mRNA expression in MNCS following whey proteins intake; *n* = 12 per group; *P < 0.05.

**Figure 5:**
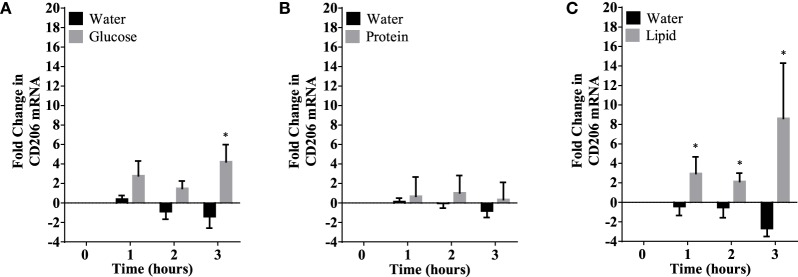
CD206 mRNA expression changes in mononuclear cells following macronutrient intake of glucose **(A)**, whey proteins **(B)**, and lipids **(C)**. Glucose intake induced significant changes at 3 h post glucose intake while lipids intake induced significant changes 1–3 h post challenge; *n* = 12 per group; *P < 0.05.

Caloric intake (300 kcal) of macronutrients (glucose, proteins, lipids) in normal volunteers changed insulin and blood glucose significantly (Figure 1). Glucose intake increased insulin concentrations at 1 and 2 h while whey proteins intake increased insulin concentrations at 1–3 h (*P < 0.05; Figures 1A,B). There were no changes in insulin concentrations following lipids intake. Whey proteins intake decreased blood glucose significantly at hours 1, 2, and 3 (*P < 0.05; Figure 1D); whereas, blood glucose concentration was decreased at 2 and 3 h following glucose and lipid intakes. Whey proteins intake resulted in a significant mRNA expression increase in CD68 and CD11b at 1, 2, and 3 h post intake (*P < 0.05; Figures 2 and 3) and mRNA downregulation of IL-6 in MNCs (*P < 0.05; Figure 4) suggesting M2 like monocyte polarization. However, there were no significant changes in CD206, CD86, CD163, CD169, CD36, CD16, and CD14 following whey proteins intake (Figure 5B; Table 2). Lipids, on the other hand, induced the expression of CD11b mRNA in MNCs (Figure 3C) and CD206 (Figure 5C). There were no significant changes in mRNA expression of CD68, IL-6, CD86, CD163, CD169, CD36, CD16, and CD14 (Figures 2 and 4; Table 2). Glucose intake changed the mRNA expression levels in MNCs of CD206 at 3 h only (*P < 0.05; Figure 5). Glucose intake did not cause any changes in the mRNA expression of the other macrophage differentiation markers examined in this study.

**Table 2:** Fold change in mRNA expression of CD86, CD163, CD169, CD36, CD14, and CD16 in mononuclear cells of normal volunteers following equicaloric intake of glucose, whey proteins, and lipids.

	Fold change in mRNA expression (mean ± SEM)	Time (h)		
		1	2	3
Glucose intake	CD86	−0.020 ± 0.184	−0.292 ± 0.120	−0.600 ± 0.234
	CD163	0.604 ± 0.598	−0.401 ± 0.498	0.556 ± 0.350
	CD169	0.621 ± 0.345	−1.128 ± 0.871	−0.078 ± 0.499
	CD36	−0.727 ± 0.59	−0.455 ± 0.314	0.218 ± 0.434
	CD14	−0.657 ± 0.387	−0.247 ± 0.164	−0.292 ± 0.241
	CD16	−0.633 ± 1.640	−1.508 ± 4.174	−0.645 ± 0.675

Whey proteins intake	CD86	0.313 ± 0.535	0.178 ± 0.412	0.332 ± 0.514
	CD163	−0.581 ± 0.569	0.113 ± 0.925	−0.771 ± 0.503
	CD169	0.295 ± 0.653	−0.315 ± 0.776	−0.130 ± −0.597
	CD36	−0.477 ± 0.496	−0.181 ± 0.424	−0.265 ± 0.526
	CD14	−0.714 ± 0.790	−0.335 ± 0.328	−0.473 ± 0.282
	CD16	−0.695 ± 0.995	2.838 ± 2.441	4.900 ± 4.560

Lipids intake	CD86	0.290 ± 0.713	−0.193 ± 0.594	0.001 ± 0.794
	CD163	0.325 ± 0.725	−0.279 ± 0.533	0.648 ± 0.739
	CD169	−0.359 ± 0.722	−2.012 ± 1.271	−0.518 ± 0.446
	CD36	−0.002 ± 0.187	−0.129 ± 0.163	0.573 ± 0.613
	CD14	−0.681 ± 0.302	−0.942 ± 0.658	0.098 ± 0.72
	CD16	−0.462 ± 0.485	−0.372 ± 2.109	1.107 ± 2.168

No changes in mRNA expression were also observed following water challenge (data not shown); *n* = 12 per group; *P < 0.05, mean ± SEM.

## Discussion

The immunomodulatory effect of macronutrients on monocytes phenotypes was observed in healthy humans. Whey proteins intake induced CD68 expression whereas the other macronutrients tested did not change CD68 expression levels. CD11b was also induced following whey proteins intake. Both CD68 and CD11b are also considered a pan-myeloid marker. CD11b is the integrin alpha M chain and is important for the adherence of neutrophils and monocytes to stimulated endothelium (18). Furthermore, CD11b plays a role in phagocytosis of complement coated particles (19). In addition, IL-6 expression was also inhibited following protein intake suggesting monocyte polarization to the M2 like phenotype. This is consistent with what is known about the protective properties of whey proteins as it promotes weight loss, reduces body fat, maintains muscle mass, reduces blood pressure, improves cardiovascular health, and contributes to satiety control (20–24). Many animal studies have reported that whey proteins intake increases glutathione levels, a potent antioxidant, which can suppress ROS generation in leukocytes (25–27). Other studies demonstrated that whey proteins decrease pro-inflammatory mediators, e.g., IL-6 and TNF-α (27–29). Whey proteins intake induced insulin release as the plasma insulin concentrations increased significantly at 1–3 h. The anti-inflammatory effects of whey proteins could be mediated through insulin release, which exerts anti-inflammatory effects in humans (13, 30–33). Glucose, on the other hand, increased insulin concentration up to 2 h and its pro-inflammatory effects probably counteracted the anti-inflammatory effect of insulin. Further studies are needed to explore this proposed mechanism.

Lipids, in the form of whipping cream, has been shown previously to generate ROS, increase serum LPS, the expression of toll-like receptor-4, and markers of inflammation such as NF-κB binding and TNFα in MNCs (15, 34, 35). The choice of whipping cream was based on the fact that it previously showed positive results and for the convenience of the subjects. Unexpectedly, our results showed that lipids did not induce M1 like monocyte phenotype, but rather induced upregulation of a pan-macrophage marker, CD11b, and a marker of M2 macrophages, CD206. These two findings suggest that there is mild polarization toward the M2 like phenotype, but not as profound as with whey proteins (which inhibited the expression of IL-6, a marker of M1). Our results might be explained by the fact that whipping cream products contains different amounts of saturated fats and monounsaturated fats, which have been shown to activate M1 and M2, respectively. Saturated fats [e.g., palmitate (PA)] have been associated with pro-inflammatory changes in adipose tissue macrophages and can activate TLR4 signaling pathways, inducing pro-inflammatory gene transcription *via* activation of NF-κB (36–39). Additionally, when PA was incubated *ex vivo* with macrophages, it resulted in elevated expression of pro-inflammatory genes (e.g., IL-6 and TNFα), characteristic of the M1 macrophage profile (40). In contrast, palmitoleate, a monounsaturated fatty acid, is secreted by adipose tissue and was shown *in vivo* to protect against the development of fatty liver and improve insulin sensitivity (41–43). Treatment of macrophages, *in vitro*, with palmitoleate or oleic acid led to the expression of anti-inflammatory M2 genes such as IL-10 (40, 44, 45). It is worth mentioning that adding palmitoleate to the macrophages that were already polarized to M1 by palmitate reversed this polarization (40) and adding palmitic acid to a culture of oleic acid-induced M2 macrophages resulted in a complete inhibition of oleic acid-induced markers of M2 cells, Arg1, and CD206 (45). Polyunsaturated fats (PUFA) can elicit different pro-inflammatory (e.g., n-6 PUFA) or anti-inflammatory (e.g., docosahexaenoic acid and eicosapentaenoic acid) profiles, depending on the type of PUFA (46–49). Accordingly, our results showed that whipping cream intake induced mild polarization toward M2 like monocytes, possibly due to the opposing polarizing stimuli. Further studies utilizing different fats are needed to dissect their effects on monocyte subsets differentiation.

In our study, glucose did not induce any of the cluster of differentiation markers measured except CD206 at 3 h. CD206 (also termed as C-type mannose receptor 1) in MNCs is considered a M2 macrophage marker (50, 51). However, CD206 function is not yet fully understood. Although it was suggested that CD206 has a role in the resolution of inflammation by clearing inflammatory molecules from the blood (36), macrophages expressing CD206 have unfavorable profibrotic effects. These macrophages promote fibroblast growth through TGF-β and chemokine production (52). Additionally, CD206 expressing macrophages may undergo a fibrocyte-like phenotype switch and produce collagen (53). Interestingly, CD206 upregulation was only observed following glucose and lipids intake. There were no changes observed on CD206 levels following whey proteins intake. Although glucose was shown to induce ROS generation and inflammation in MNCs in multiple studies (13), our study showed the lack of M1 surface markers after glucose intake. This unexpected finding, however, is similar to a recent study by Kratz et al. that identified a distinct population of adipose tissue metabolically activated macrophages (MMe) following glucose intake in obese mice and humans (54). In detail, their findings demonstrated that when macrophages are treated with glucose, palmitate, and insulin (metabolic activation), this produces a unique macrophage phenotype that is mechanistically distinct from classically activated macrophages (M1), suggesting that the metabolic activation drives macrophage polarization *via* mechanisms that are different from the classic activation. These cells do not express the classic markers of M1 or M2 macrophages, suggesting that they might express different phenotypical markers when metabolically stimulated (by glucose or palmitate). Further studies are needed to explore the surface markers on monocytes following glucose challenge.

Human monocytes in circulation have been classified into three subsets based on the expression of the surface markers CD14 and CD16 (1). However, macronutrients intake did not change the expression levels of CD14 and CD16 in human monocytes significantly suggesting the polarization of monocytes to different subsets of monocytes than the classical and non-classical monocytes characterized by CD14 and CD16. The characterization of these subsets of human monocytes is still ill-defined and further studies are needed to elucidate their characteristics. One of the limitations of our study is that it was performed on healthy subjects only. Future studies on the obese population may yield different results as obesity is associated with higher levels of subclinical inflammation and is associated with insulin resistance. Furthermore, MNCs represent a mixture of cells (monocytes, B cells, and T cells). This heterogeneity of cells utilized in this study represents a significant shortcoming in this study as large quantities of blood are needed to conduct such studies. Characterization of pure human monocyte phenotypes following macronutrients intake would be a better approach.

## Conclusion

Macronutrients induce different monocytes subtypes in human circulation with different phenotypical markers. A distinct population of MMe-like monocytes may appear in human circulation following different macronutrient intake. Whey proteins polarize monocytes to M2-like characteristics, evident by the higher levels of CD68 and CD11b and lower levels of IL-6. Lipids intake polarizes monocytes to another subset with higher levels of CD206 and CD11b. Glucose, on the other hand, induces monocytes subsets with higher levels of CD206. Examination of other macrophage differentiation markers following macronutrients intake is needed to further characterize monocytes subsets that result from different macronutrients intake. Studying the effects of the immunomodulatory effects of different macronutrients on monocyte phenotypes could open new possibilities for the identification of different nutrients controlling the metabolic activation of immune cells.

## Ethics Statement

The proposal has been reviewed and approved by the Institutional Review Board (IRB) at King Abdulaziz Medical City with an IRB number SP15/026 on the 15th of June 2015.

## Author Contributions

All authors contributed extensively to the manuscript and assumed full responsibility for its content. AALS, MD, and AALJ conceived the proposal study design and analyzed data and contributed to the writing of this manuscript. AK, MA, AALS, and HS designed and performed experiments, analyzed data, and assisted in paper writing. MZ and AN supervised the project and performed several of the assays in this study. MZ and YA assisted in sample collection and developed and executed several assays. All authors assisted in manuscript preparation.

## Conflict of Interest Statement

The authors declare that the research was conducted in the absence of any commercial or financial relationships that could be construed as a potential conflict of interest.
